# Salivary inflammatory markers and microbiome in normoglycemic lean and obese children compared to obese children with type 2 diabetes

**DOI:** 10.1371/journal.pone.0172647

**Published:** 2017-03-02

**Authors:** Waleed F. Janem, Frank A. Scannapieco, Amarpeet Sabharwal, Maria Tsompana, Harvey A. Berman, Elaine M. Haase, Jeffrey C. Miecznikowski, Lucy D. Mastrandrea

**Affiliations:** 1 Department of Pediatrics, Jacobs School of Medicine and Biomedical Sciences, University at Buffalo, Buffalo, NY, United States of America; 2 Department of Oral Biology, School of Dental Medicine, University at Buffalo, Buffalo, NY, United States of America; 3 Department of Epidemiology and Environmental Health, School of Public Health and Health Professions, University at Buffalo, Buffalo, NY, United States of America; 4 Department of Pharmacology and Toxicology, Jacobs School of Medicine and Biomedical Sciences, University at Buffalo, Buffalo, NY, United States of America; 5 Department of Biostatistics, School of Public Health and Health Professions, University at Buffalo, Buffalo, NY, United States of America; Medical University of South Carolina, UNITED STATES

## Abstract

**Background:**

There is emerging evidence linking diabetes with periodontal disease. Diabetes is a well-recognized risk factor for periodontal disease. Conversely, pro-inflammatory molecules released by periodontally-diseased tissues may enter the circulation to induce insulin resistance. While this association has been demonstrated in adults, there is little information regarding periodontal status in obese children with and without type 2 diabetes (T2D). We hypothesized that children with T2D have higher rates of gingivitis, elevated salivary inflammatory markers, and an altered salivary microbiome compared to children without T2D.

**Methods:**

Three pediatric cohorts ages 10–19 years were studied: lean (normal weight—C), obese (Ob), and obese with T2D (T2D). Each subject completed an oral health survey, received a clinical oral examination, and provided unstimulated saliva for measurement of inflammatory markers and microbiome analysis.

**Results:**

The diabetes group was less likely to have had a dental visit within the last six months. Body mass index (BMI) Z-scores and waist circumference/height ratios were similar between Ob and T2D cohorts. The number of carious lesions and fillings were similar for all three groups. The gingival index was greater in the T2D group compared to the Ob and C groups. Although salivary microbial diversity was minimal between groups, a few differences in bacterial genus composition were noted.

**Conclusions:**

Obese children with T2D show a trend toward poorer oral health compared to normal weight and obese children without T2D. This study characterizes the salivary microbiome of children with and without obesity and T2D. This study supports a modest link between T2D and periodontal inflammation in the pediatric population.

## Introduction

Obesity is common in the US adolescent population, with its prevalence tripling over the past four decades [1, 2]. Furthermore, type 2 diabetes (T2D) in children and adolescents is increasing in parallel with increasing rates of obesity [3]. While type 1 diabetes represents >90% of the new diabetes diagnoses in children, recent data demonstrates that the incidence of T2D is equivalent in at-risk pediatric populations [4], such as those of minority race/ethnicity and strong family history of T2D. Both obesity and T2D confer significant morbidity and mortality. Obesity is strongly correlated with metabolic syndrome, which is characterized by insulin resistance, glucose intolerance, dyslipidemia, and hypertension [5]. Chronic low-grade inflammation develops in the obese state as a result of a complex interaction between adipocytes and immune cells that infiltrate adipose tissue. The secreted cytokines and adipocytokines contribute to the pathogenesis of insulin resistance and metabolic disease and are also implicated in risk of oral disease such as dental caries, gingivitis and periodontitis [6, 7].

There is emerging evidence in adults for a bidirectional relationship between diabetes and periodontitis, a biofilm-induced inflammatory disease that ultimately destroys the connective tissue and bone supporting the teeth [8]. Periodontal disease may worsen glycemic control in T2D, and individuals with poor glycemic control tend to have more severe dental disease [9, 10]. While there is also some evidence that therapies aimed at reducing the inflammatory burden of periodontal disease moderately improve glycemic control [9, 11–13], a recent randomized trial in adults using non-surgical periodontal therapy demonstrated no change in hemoglobin A1c (HbA1c) in spite of improvement in periodontal measures [14]. Nevertheless, while treatment of periodontal disease may not improve overall glycemic control, preventive dental care could have a positive impact on the adverse metabolic consequences of T2D.

There has been a recent surge in the characterization of the oral microbiome in health and periodontal disease. Specific organisms within the oral cavity are associated with initiation and progression of periodontal disease [15]. In addition, alterations in the oral microbiome, termed dysbiosis, have been identified that may initiate and/or contribute to the progression of a variety of inflammatory states and chronic diseases, including atherosclerosis and cardiovascular disease, both of which are increased in T2D [5]. In fact, infection of ApoE null hyperlipidemic mice with four established periodontal pathogens–*Porphyromonas gingivalis*, *Treponema denticola*, *Tannerella forsythia*, and *Fusobacterium nucleatum*, led to elevations of serum inflammatory markers and progression of atherosclerotic plaque lesions [16]. These observations highlight the importance of a better understanding of the relationship between the oral microbiome and chronic inflammation associated with obesity and T2D.

Several studies show higher rates of caries and gingivitis in children with type 1 diabetes [17, 18], yet there is a relative absence of information regarding the oral health status of obese children with and without T2D [19]. Gingivitis is inflammation of the gingiva (gums). In some patients, gingivitis progresses to periodontitis, which results from destruction of the periodontal ligament and alveolar bone supporting the teeth. Many studies have reported that selected inflammatory biomarkers are elevated in patients with periodontal disease and diabetes [20–22]. Biomarkers detected in saliva, possibly derived from serum or directly released into saliva from epithelial and inflammatory cells in the crevicular space, may reflect the systemic and/or oral inflammatory status of the subject. Inflammatory biomarkers that have been found to be elevated in the saliva of adult individuals with T2D and periodontal disease include interleukin 1-β (IL-1β) and C-reactive protein (CRP) [22–24]. To our knowledge, there are no published studies of salivary biomarkers and the oral microbiome in adolescents with and without T2D. For effective preventive care in T2D adolescent patients, accurate understanding of the oral immuno-microbiologic state and early diagnosis is essential. This study examines the oral health, salivary biomarkers, and the oral microbiome of obese children with normal and abnormal glucose tolerance. We hypothesized that children with T2D have higher rates of gingivitis (a pre-periodontal condition), elevated salivary inflammatory markers, and an altered salivary microbiome compared to children without T2D.

## Materials and methods

### Study population

This cross-sectional study was approved by the Children and Youth’s Institutional Review Board a University at Buffalo and was conducted in accordance with the Helsinki Declaration. Subjects were recruited from the Diabetes Center and Endocrine Clinics at Women and Children’s Hospital of Buffalo over a period of two years (Feb 2013 to Feb 2015). The subject’s parent/guardian provided signed informed consent with assent or consent provided by the subject.

### Inclusion criteria

Male and female children 10–19 years of age of all racial and ethnic groups were included. Subjects with body mass index (BMI) ≤ 85^th^% for age and gender (normal weight) were included in the control group (C), subjects with BMI ≥ 95^th^% for age and gender without T2D were included in the obese group (Ob), and subjects with BMI ≥ 95^th^% for age and gender with diagnosis of T2D were included in the diabetes group (T2D).

### Exclusion criteria

Individuals with antibody-positive diabetes consistent with T1D, steroid-induced or cystic fibrosis-related diabetes, antibody-negative diabetes consistent with Monogenic Diabetes of Youth (MODY), and chronic inflammatory diseases were excluded. We also excluded individuals who used daily ibuprofen or aspirin, oral or inhaled glucocorticoids, antibiotics with the last 3 months, or atypical anti-psychotic drugs. Individuals were not included if they had a history of rheumatic fever, required antibiotic prophylaxis prior to dental treatment, history of a bleeding disorder, any recent tooth extraction (within 1 month), history of oral candidiasis, or an acute illness with fever, vomiting, diarrhea within 5 days of the study. We did not include incarcerated individuals or subjects living in group homes.

### Study measurements

For all subjects, the following information was collected in one visit before 11AM: a) anthropometry including height (Ht; nearest 0.1 cm), b) weight (nearest 0.1 kg), c) waist circumference (WC; nearest 0.1 cm), d) blood pressure, and e) HbA1c for Ob and T2D groups. Height was measured using a calibrated wall-mounted stadiometer and weight was measured with an electronic scale. BMI was calculated as kg/m^2^ and BMI Z-scores for age and sex were determined [25]. With the subjects standing, waist circumference was measured at the top of the iliac crest after gentle exhalation. It was measured using a flexible linen tape, and average of triplicate measures was collected for analysis. Further, the Ht/WC ratio was calculated for each subject. Blood pressure was measured by trained nurses using a Welch Allyn Spot Vital Signs monitor with the patient in a seated position. HbA1c was measured using Siemens DCA Vantage Analyzer.

### Oral health survey and clinical dental exam

Subjects completed an oral health survey regarding accessibility to dental health professionals and dental hygiene home-care regimen (Dental Survey in S1 Supporting Information). After refraining from eating, drinking and oral hygiene for at least 1 hour, subjects rinsed their mouths with water and then provided an unstimulated salivary sample (5-10mL) in a sterile 50mL tube. Subjects were allowed up to 30 minutes for collection of saliva. Salivary samples were kept on ice until processing. After saliva collection, all subjects underwent a modified clinical dental exam. The number of present, decayed, missing, and filled teeth (DMFT) were recorded. Index teeth [3, 9, 12, 19, 25 and 28] according to the universal numbering system for permanent dentition adopted by the American Dental Association [26] were assessed using the gingival index [27] score as follows: a score of 0 was assigned to gingiva showing a healthy pink color with no bleeding on probing; a score of 1 was assigned where gingiva was found to be red with no bleeding on probing; a score of 2 was assigned where the gingiva was red and found to bleed on probing; a score of 3 was assigned where severe inflammation/ulceration and profuse/spontaneous bleeding was noted. Subjects were also assigned a gingival rating based on the gingival index score where 0 = excellent, 0.1–1.0 = good, 1.1–2.0 = fair, and 2.1–3.0 = poor. A manual periodontal probe was used to assess pocket depth at four sites (mesiobuccal, distobuccal, mesiolingual, and distolingual) for each of the index teeth. The pocket depths for all index teeth were averaged to give an overall pocket depth score. A single examiner performed all examinations (WFJ).

### Salivary sample processing and analysis for inflammatory markers

Saliva was processed within one hour of collection. Protease inhibitor cocktail (Sigma Aldrich, Atlanta, GA) 2 μL/mL of saliva and sodium orthovanadate (400mM, Sigma Aldrich, Atlanta, GA) 3 μL/mL of saliva was added to each sample that was then centrifuged at 4°C and 1500xg for 15 minutes. Saliva samples were then divided into 0.5 mL aliquots and frozen at -80°C until assayed. The pellet collected during centrifugation was frozen at -80°C until processed for microbiome analysis (see below).

Salivary samples were assayed for acidity using short-range pH paper (range 5.5–8.0 with 0.2 increments). Salivary glucose levels were measured using a Bayer Contour Next glucometer (Bayer Healthcare, Mishawaka, IN). The detection limit of the meter is 10 mg/dL. Duplicate samples were assayed for nitric oxide using Griess reagent [28] and for CRP and IL-1β by ELISA (Salimetrics LLC, State College, PA). Lower limits of detection for ELISA were 0.43 pmol/L for CRP and 0.022 pmol/L for IL-1β.

### Microbiome analysis

The pellet obtained following centrifugation of the salivary samples was resuspended in 300 μl of lysis solution (20 mg/mL lysozyme in 20 mM Tris-HCl, pH 8.0; 2 mM EDTA; 1.2% Triton X-100) and incubated at 37°C for 30 min. Viscous samples were dissociated by adding 20 μl of 1M DTT to 300 μl of lysozyme solution, vortexed, and incubated at 56°C for 15 minutes. Salivary DNA was extracted using the QIAsymphony SP Complex-200_V6_DSP protocol [29]. Amplicon PCR was performed for the 16S rRNA hypervariable region V3-V4 using forward V3 (5' CCTACGGGNGGCWGCAG 3') and reverse V4 primers (5' GACTACHVGGGTATCTAATCC 3’). This method has been previously described as an accurate and efficient experimental approach for characterization of the complex oral microbiota [30]. Constucted libraries were sequenced on the Illumina MiSeq using lllumina reagent kit V3 chemistry to obtain paired-end (2x300bp) reads. Raw reads were processed using the Generate FASTQ workflow to obtain two fastq files (one each for forward and reverse reads) per sample [31]. DNA extraction, amplicon PCR, library construction, sequencing and quality filtering were performed at the University at Buffalo Genomics and Bioinformatics Core (Buffalo, NY).

### Microbiome data processing

Paired-end fastq reads were processed using the MiSeq workflow with the MOTHUR pipeline as previously described [32]. Briefly, taxonomic assignments were determined by using a naive Bayesian classifier with the Human Oral Microbiome Database (HOMD) reference data files (version 14.5) [33] and a 98% bootstrap confidence threshold. Phylotype clustering was carried out to bin sequences and for taxonomic classification. Shared and consensus taxonomy files at various taxonomic levels and associated metadata were used for statistical analysis.

### Statistical analysis of clinical and immunology data

Sample size was calculated *a priori* using published data comparing gingival index in obese to normal weight children [34]. Based on this data, we estimated that 17 control and 17 obese subjects with and without T2D would give our study greater than 80% power with a Type 1 error probability of 0.05 to detect a reasonable (0.99 standard deviation effect size) difference in gingival disease between normal weight and obese children with and without T2D. Data is represented as mean ± standard deviation (SD) or as percent of cohort. Data were compared using linear regression analysis for continuous variables and Fisher’s exact test for categorical variables as appropriate. P-values < 0.05 were considered as statistically significant. All statistical analyses were performed using R [35].

### Statistical analysis of microbiome data

Bray-Curtis PCoA ordinations and multi-response permutation procedure (MRPP) analyses based on non-metric multidimensional scaling (NMDS) with Bray-Curtis distances were conducted using the phyloseq library [35, 36] in R. Alpha-diversity plots were generated by the R package phyloseq, and Kruskal-Wallis tests were used to test the distribution of alpha diversity measures across the three tested groups. The Bonferroni correction was used to control the family-wise error rate at 0.05 across tests with different alpha diversity measures. With five different measures, the required significance was p-value < 0.05/5 = 0.01.

Genus-level Operational Taxonomic Units (OTUs) were assessed for differential bacterial abundance between control, obese, and T2D groups with and without adjusting for gingival index. A likelihood ratio test for the appropriate coefficients in a negative binomial, generalized log-linear model (GLM) was applied to the genus level OTU counts via the edgeR package [37]. The negative binomial dispersion parameter for each genus was estimated using an empirical Bayes estimator (see edgeR package). Genus level OTUs were filtered such that a genus without at least one count in each of C, Ob, and T2D groups was removed from testing. This procedure resulted in a matrix comprising 104 genera. The likelihood ratio tested the condition that the mean genus abundances were equal in each group with and without adjusting for gingival index in the GLM. The false discovery rate across OTUs was controlled separately in the unadjusted and adjusted analysis at 0.05 via a Benjamini and Hochberg procedure [38].

## Results

A total of 49 subjects were enrolled in the study: 16 with T2D, 14 Ob, and 19 with normal weight (C). Baseline characteristics of the subjects are summarized in Table 1. Age distribution and oral hygiene methods were similar among the three groups. Race and gender were different between the C and T2D groups, with the T2D group having more African-American and female subjects (p < 0.03 for both gender and race). This difference was not observed between Ob and the other two groups. More individuals in the Ob and T2D group carried Medicaid insurance for dental care compared to C subjects. Individuals with T2D were less likely to have had a dental visit within the last 6 months compared to the other two cohorts. The BMI Z-score and WC/Ht ratio were not different between the Ob and T2D groups (Table 2). As expected, HbA1c was significantly higher in T2D group compared to Ob group (p < 0.001).

**Table 1:** Characteristics and dental survey results for subjects by cohort. Data was collected from responses to an oral health survey (Dental Survey in S1 Supporting Information) administered to all subjects.

	Normal Wt (n = 19)	Obese (n = 14)	T2D (n = 16)	p-value
Age (years)	14.8±2.4	14.5±2.2	16.0±2.6	0.183
Gender (Female %)	37	50	81	0.028*
Race (%)				
Non-Hispanic White (n = 18)	63%	29%	12%	
African American (n = 17)	15%	29%	63%	0.022^‡^
Hispanic (n = 8)	11%	21%	19%	
Other (n = 6)	11%	21%	6%	
Dental Insurance (%)				
Commercial	74%	29%	38%	
Medicaid	32%	71%	62%	0.012^§^
Other	5%	0%	0%	
Daily brushing (%)	100%	100%	100%	
Daily flossing (%)	21%	43%	19%	0.306
Daily rinsing (%)	26%	14%	31%	0.643
Dental appointment within 6 months (%)	84%	86%	38%	0.005^||^

Post Hoc Analysis p-values.

* Normal Wt vs. Obese p = 0.497; Normal Wt vs. T2D p = 0.028; Obese vs. T2D p = 0.121.

‡ Normal Wt vs. Obese p = 0.275; Normal Wt vs. T2D p = 0.023; Obese vs. T2D p = 0.323.

§ Normal Wt vs. Obese p = 0.011; Normal Wt vs. T2D p = 0.012; Obese vs. T2D p = 0.709.

|| Normal Wt vs. Obese p = 1.000; Normal Wt vs. T2D p = 0.005; Obese vs. T2D p = 0.011.

**Table 2:** Cohort BMI status and glycemic control.

	Normal Wt	Obese	T2D	p-value
	(n = 19)	(n = 14)	(n = 16)	
BMI Z-score	-0.32±0.81	2.32±0.45	2.30±0.53	< 0.001^#^
WC/Ht	0.44±0.039	0.69±0.080	0.70±0.095	< 0.001^##^
HbA1c,%	N/A	5.24±0.22	8.64±2.63	<0.001
(mmol/mol Hb)		(33.7±2.37)	(70.9±28.8)	

WC/Ht: Waist Circumference/Height ratio, HbA1c = glycosylated hemoglobin. Post Hoc Analysis p-values.

# Normal Wt vs Obese p< 0.001; Normal Wt vs. T2D p < 0.001; Obese vs. T2D p = 0.909.

## Normal Wt vs Obese p< 0.001; Normal Wt vs. T2D p < 0.001; Obese vs. T2D p = 0.582.

There were no differences in the number of decayed, missing and filled teeth between groups (Table 3). There is a clear relationship between gingival index and group status with gingival index being significantly higher in the T2D cohort compared to the C group (Table 3, p = 0.010). Thus, the microbiome analysis across cohorts included an adjustment for gingival index (see below). The gingival ratings were concordant with the gingival index with the worst being in T2D group. More C and Ob subjects had excellent or good gingival rating when compared to T2D subjects. In fact, none of the subjects with T2D had an excellent gingival rating. Individuals who did not have a dental visit in the last 6 months had a higher gingival index (p < 0.01). Other periodontal disease outcomes, including average pocket depth, deepest pocket depth and number of pockets ≥ 4 mm, were not significantly different between groups.

**Table 3:** Dental examination findings by cohort.

Dental Findings	Normal Wt (n = 19)	Obese (n = 14)	T2D (n = 16)	p-value
Fillings/Cavities (%)				
0–1	84%	79%	75%	
2–4	5%	14%	19%	0.831
>4	11%	7%	6%	
Gingival Index	0.56±0.40	0.71±0.51	1.10±0.56	0.010^‡^
Gingival Rating (%)^†^				
Excellent	10%	14%	0%	
Good	74%	79%	50%	0.040^§^
Fair	16%	7%	50%	
Average pocket depth (mm)*	2.60±0.61	2.60±0.54	3.02±0.64	0.059
Deepest pocket depth (mm)	3.74±0.81	3.57±0.76	4.31±1.01	0.053
Number of pockets ≥4 mm	3.11±3.80	3.14±3.70	6.38±7.53	0.141

Post Hoc Analysis p-values.

‡ Normal Wt vs. Obese p = 0.362; Normal Wt vs. T2D p = 0.003; Obese vs. T2D p = 0.068.

§ Normal Wt vs. Obese p = 0.856; Normal Wt vs. T2D p = 0.040; Obese vs. T2D p = 0.014.

* mm: Millimeter.

† Gingival Rating: 0, = excellent; 0.1–1.0 = good; 1.1–2.0 = fair; 2.1–3.0 = poor.

Subjects provided an unstimulated saliva sample in order to quantify several biomarkers for inflammation. Results from salivary inflammatory markers are summarized in Table 4. Salivary glucose was low in all three groups. CRP levels were similar among groups. While there was no difference in pH, nitric oxide, or IL-1β levels between groups, there was a trend (although not statistically significant) towards more acidic saliva, and higher nitric oxide and IL-1β concentrations in saliva collected from subjects with T2D.

**Table 4:** Salivary inflammatory markers by cohort.

	Normal Wt	Obese	T2D	p-value
Inflammatory Marker	(n = 19)	(n = 14)	(n = 16)	
Acidity (pH)	7.23±0.15	7.10±0.15	7.05±0.35	0.075
CRP* (pmol/L)	616.47±1478.67	570±1202.10	250.13±259.47	0.604
Nitric Oxide (pmol/μL)	90.89±35.72	98.07±34.05	113.50±37.54	0.183
IL-1β* (pmol/L)	8.32±10.85	9.82±9.42	11.59±11.12	0.662
Glucose (mg/dL)	10 ± 0	10 ± 0	11±6	0.28

*CRP: C-Reactive Protein, IL-1β: Interleukin 1 beta.

**Fig 1:**
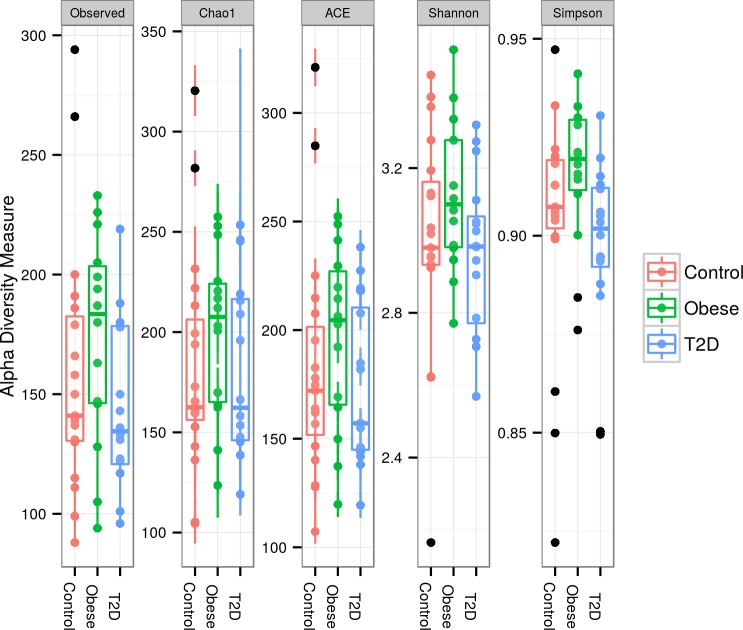
Alpha diversity as measured using five metrics. Calculations and plots were generated using the Phyloseq package. Unadjusted p-values using the Kruskal-Wallis test for microbiome alpha diversity measures were as follows: Observed: 0.1594; Chao: 0.2760; Ace: 0.2872; Shannon: 0.1714; and Simpson: 0.0354. No significant differences at p<0.01 were observed between groups.

In order to determine if oral bacterial populations differ among the three cohorts, DNA extracted from the salivary pellet was used to sequence the V3-V4 hypervariable region of the 16S rRNA. Approximately 3.2 million reads were obtained after quality filtering, an average of approximately 66 thousand reads per sample. Phylotypes were assigned based on the Human Oral Microbiome Database (HOMD) [39]. We first compared the alpha diversity between cohorts, a diversity metric which evaluates both the richness and evenness of a population. Alpha diversity measures between the C, Ob, and T2D subjects using five different metrics are shown in Fig 1. In general, although the alpha diversity scores tended to be higher for the obese group and lower for the control and T2D groups across all metrics, there was no significant difference in alpha diversity among groups. The borderline univariate Simpson significance (Fig 1; p-value < 0.04) is due to a significant difference between the Ob and T2D groups. However, a Bonferroni family-wise error control at 0.05 where each test would need p<0.01 for significance yielded no significant alpha diversity differences between these two groups.

**Fig 2:**
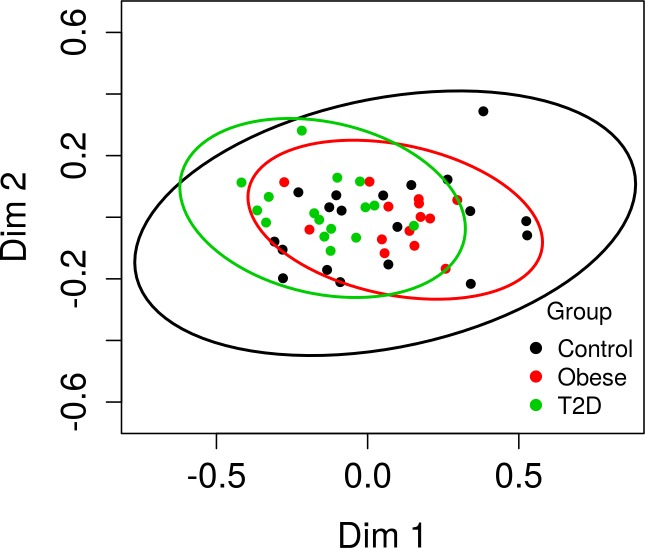
Bray Curtis based NMDS plots of all samples. Ellipsoids represent 95% confidence intervals surrounding each cohort. MRPP analysis indicates that control group members are more dissimilar than expected by chance (agreement statistic A = 0.049; p<0.001).

To assess group similarities in species composition as a measure of beta diversity, a multi-response permutation procedure (MRPP) analysis was employed using the Bray-Curtis distance matrix. To visualize the results, a non-metric multidimensional scaling (NMDS) plot is shown in Fig 2. Bray-Curtis index provides a measure of community/species composition differences between samples based on OTU counts, regardless of taxonomic assignment. Ordinations based on this metric demonstrate no obvious ‘location’ separation between groups; however, there is a ‘spread’ difference (agreement statistic A = 0.049, p <0.001) due to the control group having a larger variation (larger 95% confidence ellipsoid). This spread difference may be due to several control samples being considered as outliers across alpha and beta diversity measures (Fig 2).

**Fig 3:**
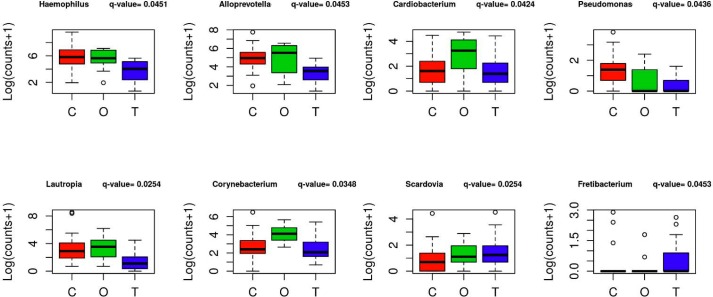
OTUs at genus level that are significantly different across groups (C = control, O = obese, T = Type 2 diabetes). The relative abundance is shown as log(counts +1) for the significant OTUs with false discovery rate controlled at 0.05 via a Benjamini and Hochberg procedure. The q-values were computed from the p-values from a likelihood ratio test applied to a Negative Binomial generalized linear model fit using the edgeR package. Note, ***Lautropia*, *Corynebacterium*, and *Cardiobacterium*** were significantly different after adjusting for gingival index.

Genus level OTUs were assessed for differential abundance using a likelihood ratio test for the appropriate coefficients in a negative binomial generalized log-linear model with significance controlled at 0.05 via a Benjamini and Hochberg scheme. This resulted in eight significantly different OTUs at the genus level as shown in Fig 3. After adjusting for gingival index, 5 genus level OTUs were significantly different, 3 of which were identified in the primary analysis–*Lautropia*, *Corynebacterium*, and *Cardiobacterium* while the 2 additional OTUs were *Lactobacillus* and *Cryptobacterium*.

## Discussion

This study examined the periodontal status of subjects 10–19 years old with normal weight, or obesity with and without T2D. Adverse health outcomes related to T2D and obesity are of increasing concern in the pediatric population. In adults with these diagnoses, there is a higher incidence of periodontal disease, as represented by gingivitis and periodontitis. Longitudinal studies also highlight an association between weight gain and new cases of periodontitis [6, 40, 41]. In adults with T2D, diabetes-related factors such as duration of diabetes, fasting blood glucose, and HbA1c correlate with periodontal health [42].

The demographic data for race and gender among our cohorts is consistent with epidemiologic data in that T2D is overrepresented in females and African-Americans [3]. Most subjects performed minimal daily dental hygiene in the form of brushing teeth twice daily as recommended by American Academy of Pediatrics [43]. However, our data suggest that children with diabetes may have less access to dental care compared to normal weight and obese subjects as demonstrated by the small number of subjects with T2D having reported a dental visit within the previous six months. This difference is not attributable to lack of dental insurance.

Although rates of missing, decayed, and filled teeth were similar between groups, the gingival index (Table 3), which assesses the severity of gingival inflammation, was increased in those with T2D compared to the C group. More severe gingival disease has also been observed in children with type 1 diabetes and may be related to elevated glucose levels that encourage proliferation of oral bacteria or aberrant host response [17, 18, 44]. While previous data showed higher salivary glucose levels in adults with T2D compared to controls [45], salivary glucose levels were not different between the groups in this study.

Pocket depth, the space between a tooth and the gingiva, is deeper in a tooth with periodontitis as the result of bone loss. Alternatively, pocket depths can become deeper as the result of edema in the gingiva around teeth without bone loss. We found no difference in pocket depths between groups. This result is not surprising since chronic gingivitis and subsequent periodontitis is rare in the pediatric and young adult population [46]. However, there was a trend towards increased average pocket depth, deepest pocket depth, and number of pockets ≥4 mm in the T2D group. These observations suggest that pediatric T2D patients are at increased risk for development of periodontitis. A larger cross-sectional study should clarify this finding. Similarly, a prospective study in this population would assist in determining progression to periodontal disease in younger groups diagnosed with T2D.

For many inflammatory markers, particularly CRP and IL-1β, there is moderate-to-strong correlation between serum and salivary levels [47, 48]. Salivary levels of IL-1β, CRP and NO levels have been reported to be higher in patients with periodontal disease when compared to healthy dentate patients [24, 49–52]. In addition, salivary and serum NO levels correlate in chronic, aggressive periodontitis [53]. There is limited data related to salivary inflammatory markers in obesity and T2D. One study showed that obesity is associated with increased salivary level of CRP [54], while another study showed that IL-1β concentration in saliva is associated with periodontal disease severity rather than diabetes [23]. In this study, we included salivary markers that have been demonstrated previously to be associated with periodontal disease, since there is no information available regarding salivary biomarkers of inflammation and periodontal disease in pediatric cohorts. Specifically, we chose CRP because it is associated with systemic inflammation related to obesity and insulin resistance [5]. IL-1β was also examined since this inflammatory cytokine has been associated consistently with severity of periodontal disease and as a stimulator of bone resorption in adults [23]. We chose nitric oxide because it is an indicator of endothelial cell damage and may constitute a nonspecific defense mechanism against oral bacteria [52]. Although we found no difference in pH or NO levels between groups, there was a trend towards a lower pH and higher NO concentrations in saliva collected from subjects with T2D. Again, due to the low prevalence of gingival disease in children [46], this difference in acidity and NO concentration might become more apparent with a larger sample size. The wide variation in salivary levels detected might also have contributed to the failure to identify statistical differences between the groups. Salivary CRP levels were similar among all three cohorts in this study. Of note, our study is limited by the fact that the C group has wide variations in salivary CRP levels, with mean salivary CRP higher than both the Ob and T2D group. While we excluded individuals with a history of chronic inflammatory diseases, we did not obtain a corollary serum CRP level in this study. A serum level would have identified individuals with acute phase reactant elevation of serum CRP that could contribute to increased salivary CRP. Studies have shown that other salivary inflammatory markers, including tumor necrosis factor- alpha, matrix metaloproteinases, interleukin-6, and interleukin-8, are associated with dental health and periodontitis [21, 22]. However, given that the oral disease burden was low in our study cohorts, measurement of these salivary markers would not necessarily strengthen the overall findings. Future studies to explore a broader range of salivary inflammatory cytokines by multiplex assays will clarify the relevance of these markers in pediatric dental disease.

The microbial flora of dental plaque biofilms associated with development of gingivitis and periodontitis in adults has been studied extensively [55]. Classical studies using the human experimental gingivitis model support the concept that bacteria in plaque induce gingivitis and that plaque removal by mechanical oral hygiene eliminates inflammation and reverses disease [56]. The major microbiota contributing to dental disease were initially identified by bacterial culture studies and later by DNA-DNA hybridization techniques, by assessing the major cultivable bacterial species from supra- and sub-gingival plaque. These culture-based techniques led to the observation that the oral microbial flora shifts from predominantly Gram-positive cocci and filaments in health, to Gram-negative anaerobes in gingivitis. Reported species in gingivitis include *Actinomyces species*, *Veilonnella parvula*, *Capnocytophaga gingivalis*, *Capnocytophaga ochracea*, *Capnocytophaga sputigena*, *Campylobacter rectus*, *Campylobacter showae*, *Fusobacterium nucleatum*, *Prevotella nigrescens*, *Leptotrichia buccalis*, *Propionibacterium acnes* and *Selenomonas noxia*. Also, studies of samples from patients with periodontitis have identified a subgroup of species, the so-called “red complex” that includes *Porphyromonas gingivalis*, *Tannerella forsythia* and *Treponema denticola* [57]. More recent molecular method techniques based 16S rRNA sequencing, which is used to define the oral microbiome, have revealed further complexities, including the observation that much of the oral flora has yet be cultivated [58], including bacteria such as *Filifactor alocis* and TM7 [59, 60]. However, the contribution of these bacteria to human oral disease is yet unclear. Additional, but often unappreciated, insight afforded by studies of the oral microbiome, developed from 16S microbiome, metagenomic and metatranscriptomic approaches, include the observation that there is considerable variation in the microflora within and between patients, including healthy and diseased sites [61, 62].

The role diabetes plays in altering the oral microbiome as it relates to periodontal disease is under continuous study. In a population of adults with no prior diagnosis of T2D, those identified with prediabetes had elevated levels of *Aggregatibacter actinomycetemcomitans*, *P*. *gingivalis*, *T*. *denticola*, and *T*. *forsythia*, while *Actinomyces naeslundii* was diminished in the subgingival biofilm [63]. Using 16S rRNA sequencing, the subgingival plaque bacterial composition was assessed in adults with and without T2D. In plaque from subjects with healthy periodontium, the abundance of *Prevotella*, *Pseudomonas*, and *Tannerella* were significantly different between individuals with and without diabetes [64]. The overall variation was not high with only 3/126 sequences classified at the genus level being different between the groups with and without diabetes. Casarin et al. demonstrated significant differences in the subgingival microbiota between adults with chronic periodontitis with and without poorly controlled T2D [65]. These results may not be directly applicable given that our cohort had minimal oral disease and better glycemic control.

The oral microbiome in patients with periodontal disease is most often studied by sampling supra- and/or subgingival dental plaque. We sought to identify microbial biomarkers of periodontal disease from saliva, an easily collected and stable biologic sample. This is the first study to describe the oral salivary microbiome of children with type 2 diabetes as compared to normal weight and obese children. No overall differences in microbial genera, as assessed using multiple alpha-diversity measures, were found among the three groups. Indeed, the non-metric multidimensional scaling (NMDS) plot shows much overlap between the groups with respect to the presence of OTUs. Some variation was noted between groups at the genus level. *Fretibacterium* was unique to the T2D group. *Cardiobacterium* and *Corynebacterium* were elevated in the Ob group as compared to both C and T2D, while *Haemophilus*, *Alloprevotella*, *Pseudomonas* and *Lautropia* were reduced in the T2D group compared to the other groups. The finding related to *Pseudomonas* is in contrast to those of Zhou et al., who identified increased abundance of *Pseudomonas* in subgingival plaque collected from subjects with type 2 diabetes [64]. Interestingly, elevated levels of *Scardovia*, a bacterium recently associated with dental caries [66], were identified in the C group. After adjusting for gingival index as a measure of oral health, three OTUs continued to vary between the groups–*Lautropia*, *Cardiobacterium*, and *Corynebacterium*. Further studies using techniques that allow for characterization of the microbiome at the species level may reveal differences to help explain disease risk.

The lack of diversity between the salivary microbiome of these cohorts may be explained by the relatively small sample size of each group. It is also possible that the young age of these subjects and the relatively shorter duration of disease does not allow for diversification of the microbial flora. It is notable, that even among adults with significant metabolic disease, the overall diversity between groups with and without T2D is relatively low (see above). This is consistent with our findings, in that we identified 8/104 OTUs that differed among the groups.

In summary, children with T2D show a trend toward poorer oral health compared to normal weight and obese children without T2D. Microbial diversity between the groups is minimal, although several differences in the relative abundance of a few bacterial genera were noted. While a relative lack of microbial diversity was noted within each group, several bacterial genera differed between groups. Further exploration of the oral microbiome and inflammatory markers will enhance our understanding of the evolving microenvironment in children with T2D as well as the potential relationship to periodontal disease.

## Supporting information

S1 Supporting Information Dental Survey. Administered to all subjects.
(PDF)
