# Correction: Salivary inflammatory markers and microbiome in normoglycemic lean and obese children compared to obese children with type 2 diabetes

**DOI:** 10.1371/journal.pone.0183600

**Published:** 2017-08-16

**Authors:** Waleed F. Janem, Frank A. Scannapieco, Amarpreet Sabharwal, Maria Tsompana, Harvey A. Berman, Elaine M. Haase, Jeffrey C. Miecznikowski, Lucy D. Mastrandrea

The third author’s name is incorrect. The correct name is: Amarpreet Sabharwal.
