# Supplementary material for: Salivary inflammatory markers and microbiome in normoglycemic lean and obese children compared to obese children with type 2 diabetes
Source: PLoS One. 2017 Mar 2;12(3):e0172647. doi: 10.1371/journal.pone.0172647 (PMC5333807; doi:10.1371/journal.pone.0172647)
Supplement: S1 Supporting Information — Administered to all subjects. (PDF) [file pone.0172647.s001.pdf]

Dental Survey

S1 Supporting Information

Patient Identifier: ___________________

Date of Birth: _______________________

Gender: ☐ Male ☐ Female

Grade in School: _____________________

Race: ☐Caucasian ☐African American ☐Asian ☐Other

Ethnicity: ☐Hispanic ☐Non-Hispanic

Insurance Status: __________________

Do you have dental insurance? ☐Yes ☐No

How would you rate the health of your child's teeth/gums?

☐Excellent ☐Very Good ☐Good ☐Fair ☐Poor ☐ Don't Know

How many times per day does your child brush his/her teeth? _________

In the last 7 days, how many times did your child floss? __________

In the last 7 days, how many times did your child use a dental rinse? _____________

At what age did your child first see a dentist? ______ years

When was the last time your child saw a dentist? ☐0-6 months ☐6-12 months ☐>12 months

How often does your child see the dentist per year? _______

How many cavities/fillings does your child have? _______

Does your child complain of pain when brushing his/her teeth? ☐Yes ☐No

Do your child's teeth bleed when brushing? ☐Yes ☐No

Does your child have any loose teeth? ☐Yes ☐No

Does your child complain of temperature sensitivity? ☐Yes ☐No

Does your child complain of tooth pain? ☐Yes ☐No

Does your child complain of gum pain? ☐Yes ☐No

Dental Survey

Has your child had braces? ☐Yes ☐No

Has the dentist told you that your child will need braces? ☐Yes ☐No

Does your child wear a retainer or other dental appliance? ☐Yes ☐No

Has your child had any teeth pulled? ☐Yes ☐No How many? _________

Has your child lost any teeth (separate from dental extraction)? ☐Yes ☐No How many? _________

Does your child have any of the following medical conditions?

Asthma	☐Yes	☐No
Diabetes	☐Yes	☐No
Food Allergies	☐Yes	☐No
Hay fever	☐Yes	☐No
Impetigo	☐Yes	☐No
Eczema	☐Yes	☐No

Does your child take any medications? ____

How many hours a night does your child sleep? __________________________________

What is your home's water supply? ☐Well Water ☐City Water

How much water does your child drink? ☐ 1-2 glasses/day ☐ 3-4 glasses/day ☐ > 5 glasses/day

Does your child (if old enough) drink alcoholic beverages? ☐Yes ☐No How many? _________

Does anyone else in your home drink alcohol? ☐Yes ☐No

Does your child smoke? ☐Yes ☐No

Does anyone else in your home smoke? ☐Yes ☐No

Does anyone in your family have any of the following?

Dental Survey

Dentures? ☐Yes ☐No

Gingivitis? ☐Yes ☐No

Periodontal Disease? ☐Yes ☐No

Do members of your household go to (check all that apply)

- ☐ One dentist
- ☐ More than one dentist
- ☐ An orthodontist
- ☐ An oral surgeon
- ☐ Other dental specialist
- ☐ Other ______________________________

The most common reason for not going to the dentist is:

- ☐ I do go for regular check-ups
- ☐ Transportation issues
- ☐ It costs too much
- ☐ I don’t need to go very often
- ☐ I forget to go
- ☐ I don’t like dentists
- ☐ There is nothing wrong with my teeth
- ☐ Other ________________________________

Dental Survey

Height (cm): ________________

Weight (kg): ________________

BMI (kg/m^2): _________________

Blood Pressure: _______________ ___________________

Waist Circumference (cm): _______________ _________________

HbA1c: ____________

Mean Blood Glucose Levels: _____________________________
